# Multi-class semantic segmentation of breast tissues from MRI images using U-Net based on Haar wavelet pooling

**DOI:** 10.1038/s41598-023-38557-0

**Published:** 2023-07-20

**Authors:** Kwang Bin Yang, Jinwon Lee, Jeongsam Yang

**Affiliations:** 1 Devision of Memory - Memory FAB Team 1, Samsung Electronics, 1 Samsungjeonja-ro, Hwaseong, Gyeonggi 18448 Republic of Korea; 2 Department of Industrial and Management Engineering, Gangneung-Wonju National University, 150 Namwon-ro, Wonju, Gangwon 26403 Republic of Korea; 3 Department of Industrial Engineering, Ajou University, 206 Worldcup-ro, Suwon, Gyeonggi 16499 Republic of Korea

**Keywords:** Breast cancer, Cancer imaging, Electrical and electronic engineering

## Abstract

MRI images used in breast cancer diagnosis are taken in a lying position and therefore are inappropriate for reconstructing the natural breast shape in a standing position. Some studies have proposed methods to present the breast shape in a standing position using an ordinary differential equation of the finite element method. However, it is difficult to obtain meaningful results because breast tissues have different elastic moduli. This study proposed a multi-class semantic segmentation method for breast tissues to reconstruct breast shapes using U-Net based on Haar wavelet pooling. First, a dataset was constructed by labeling the skin, fat, and fibro-glandular tissues and the background from MRI images taken in a lying position. Next, multi-class semantic segmentation was performed using U-Net based on Haar wavelet pooling to improve the segmentation accuracy for breast tissues. The U-Net effectively extracted breast tissue features while reducing image information loss in a subsampling stage using multiple sub-bands. In addition, the proposed network is robust to overfitting. The proposed network showed a mIOU of 87.48 for segmenting breast tissues. The proposed networks demonstrated high-accuracy segmentation for breast tissue with different elastic moduli to reconstruct the natural breast shape.

## Introduction

The International Agency for Research on Cancer has reported that breast cancer is one of the most prevalent cancers worldwide, accounting for 11.7% of all cancer cases^1^. Due to the increasing rates of breast cancer and therefore of mastectomy, the demand for breast reconstruction surgery is continuously increasing. In the preparation stage for breast reconstruction surgery, it is necessary to obtain the natural breast shape in a standing position before mastectomy. However, plastic surgeons can access only magnetic resonance imaging (MRI) or computed tomography (CT) images taken with a patient lying in a prone position during the examination process. Consequently, they are limited in producing a natural-shaped breast implant in the standing position solely from images in a prone position. To overcome this limitation, studies have been conducted on reconstructing breast shapes in the standing position from prone-position MRI images by obtaining an approximate solution through an ordinary differential equation of the finite element method for deformations caused by the center of gravity acting on the breast^2,3^. However, it is difficult to obtain meaningful results from these studies for the natural breast shape in a standing position because elastic moduli of breast tissues such as skin, fat, and fibro-glandular tissue affected by gravity are different from every other.

To address this issue, this study proposed a deep learning network using U-Net based on Haar wavelet pooling to segment breast tissues for reconstructing the breast shape in a standing position. To train the deep learning network, we constructed a dataset consisting of background, skin, fat, and fibro-glandular tissues from MRI breast images. For labeling the dataset, the median filter, Otsu’s threshold algorithm, and a template-based segmentation method were used. In the subsampling stage of the conventional U-Net, weak information about the breast tissue may be lost as the max pooling is sensitive to overfitting. This may cause a significant error during data segmentation. To improve the segmentation accuracy of breast tissues, we utilized the Haar wavelet pooling instead of the max pooling to be robust for overfitting. The U-Net based on Haar wavelet pooling simultaneously uses a low–low (LL) sub-band that holds approximate values of the input image and three distinct frequency sub-bands [low–high (LH), high–low (HL), and high–high (HH)] with detailed edge feature information. Therefore, it can effectively extract features of breast tissues by reducing the loss of image information in the subsampling stage. It also implements a deep learning network that is robust to overfitting.

Various experiments with max pooling and average pooling were conducted to compare the performance of the proposed network and other networks described in previous studies for segmenting breast tissues. The proposed U-Net based on Haar wavelet pooling achieved a mean intersection over union (mIoU) of 87.48, which was higher than those of other methods. Segmented images not only provide basic information needed to reconstruct 3D breast shapes but also help plastic surgeons make a more accurate diagnosis.

## Literature review

### Deep learning-based breast tissue segmentation method

Currently, we are observing noteworthy advances in deep learning-based image segmentation and detection for ultrasound images, CT images, and MRI^4–6^. Furthermore, deep learning research based on medical imaging is being utilized in various fields including the heart, lungs, brain, and breast^7^. Nonetheless, the segmentation of breast MRI images is advancing at a slower pace. Previous studies on breast image analysis have used binary segmentation methods to diagnose breast diseases such as breast cancer and breast tumors^8–10^. However, such methods were time-consuming and labor-intensive because the region of interest (ROI) for the breast tissue was manually set. Moreover, these methods had a disadvantage in that the quality of segmented tissues varied depending on the skill level of the worker and the algorithm used. Recently, various deep learning-based algorithms have been introduced for medical image processing to overcome such disadvantages.

Table 1 summarizes segmentation methods for breast tissues based on deep learning with MRI breast images used in previous studies. Most deep learning-based networks that segment breast tissues modified the U-Net^17^ to perform segmentation for a single class, such as breast cancer, breast tumor, and breast density. To detect breast cancer in digital mammography, Soulami et al. have improved the segmentation performance by proposing a deep learning network based on the end-to-end U-Net method^11^. Further, to segment tumors in breast ultrasound images, Negi et al. have used a deep learning network called RDA-U-Net and the Wasserstein GAN algorithm and reported remarkable performance^12^. Ilesanmi et al. have proposed a variant-enhanced block that combines max pooling and average pooling to segment tumors in breast ultrasound images, consequently improving the accuracy of semantic segmentation for tumors using the VEU-Net network^13^.

**Table 1:** Comparison of deep learning-based segmentation methods for breast tissues in existing studies.

Related study	Backbone model	Input images	# of segment classes including background	Segmented tissue
Soulami et al.^11^	End-to-end U-Net	Mammogram	2	Breast cancer
Negi et al.^12^	RDA-U-Net	Ultrasound	2	Breast tumor
Ilesanmi et al.^13^	VEU-Net		2	Breast tumor
Zhang et al.^14^	U-Net	MRI	3	Fat, fibro-glandular tissue
Zhang et al.^15^	U-Net (transfer learning)		3	Fat, fibro-glandular tissue
Huo et al.^16^	nnU-Net		3	Fat, fibro-glandular tissue
Ours	Haar wavelet pooling U-Net	MRI	4	Skin, fat, fibro-glandular tissue

Many studies have been conducted to segment fibro-glandular tissues known to account for a large proportion of breast tissues using deep learning. Zhang et al. have segmented fat and fibro-glandular tissues from MRI breast images using a U-Net^14^. Subsequently, Zhang et al. have improved the segmentation accuracy by performing transfer learning for fat and fibro-glandular tissues in a deep learning model that segments breast density of MRI breast images^15^. Huo et al. have improved the segmentation accuracy of fibro-glandular tissues by adopting nnU-Net to segment the entire breast and fibro-glandular tissues in DCE-MRI breast images^16^.

In contrast to most previous studies that used binary segmentation methods, our study performed multi-class semantic segmentation through U-Net based on Haar wavelet pooling to segment various types of breast tissues for breast shape reconstruction.

### Deep learning method based on wavelet pooling for images

The wavelet pooling used in the sampling operation of deep learning algorithms has the advantage of decreasing the effect of noise on segmentation by filtering the input image before sampling. Previous studies have conducted various image segmentation tasks by combining wavelet pooling and deep learning^18,19^. Table 2 summarizes improved performances of image classification, segmentation, recognition, and restoration using wavelet pooling-based deep learning in previous studies.

**Table 2:** Comparison of deep learning methods based on wavelet pooling for images.

Related study	Deep learning purpose	Wavelet transform	Deep learning type	Image dataset
Liu et al.^20^	Image fusion	Discrete wavelet transform	CNN	COCO
Liu et al.^21^	Image restoration	Multi-level wavelet transform	Multi-level wavelet CNN	Berkeley segmentation dataset, DIV2K, Waterloo exploration database
Li et al.^22^	Image classification	Discrete wavelet transform	CNN	MNIST, CIFAR-10, SHVN, KDEF
Suryanarayana et al.^23^	Super resolution	Stationary wavelet transform	VDR-Net	Brain MRI (IXI-MR dataset)
Alijamaat et al.^24^	Semantic segmentation	Discrete wavelet transform	U-Net	Brain MRI (MICCAI dataset)
Zhao et al.^25^	Semantic segmentation	Multi-scale wavelet transform	WU-Net	Pediatric echocardiographic (CAMUS dataset)
Ours	Semantic segmentation	Discrete wavelet transform	Haar wavelet pooling U-Net	Breast MRI (TCIA breast diagnosis)

Previous studies have proposed an unsupervised image fusion algorithm for image restoration and classification that combines a deep learning network with a multi-scale discrete wavelet transform^20–22^. Suryanarayana et al. have converted low-resolution MRI images into high-resolution MRI images by combining VDR-Net with wavelet pooling that uses both low and high frequencies^23^. Alijamaat et al. have combined U-Net with wavelet pooling of low-frequency component (LL band) characterized by high image pixel concentration while maintaining the overall trend of images to improve the segmentation performance for multiple sclerosis of the brain^24^. Zhao et al. have improved the performance of congenital heart disease diagnosis in pediatric echocardiography images by combining low-frequency information of multi-scale wavelet with WU-Net^25^.

Existing studies cited above have demonstrated that the combination of wavelet pooling and deep learning algorithms can improve image performance in various applications such as classification, segmentation, recognition, and restoration. This study improves the semantic segmentation performance for breast tissues by combining Haar wavelet pooling with U-Net. In particular, the image output through the high-frequency filter of wavelet pooling can effectively express locations of breast tissues and micro-soft tissues by emphasizing edge features for vertical, horizontal, and diagonal components. Furthermore, the characteristics of fine breast tissues are well-preserved because the image output through the low-frequency filter has approximate values of the input image.

## Design of deep learning network for segmentation

### Overview

This paper proposed a multi-class semantic segmentation method for breast tissues to reconstruct breast shapes in a standing position using U-Net based on Haar wavelet pooling. Labeled breast tissue data are necessary to train deep learning networks. MRI images, which are essential for breast cancer screening, have higher resolution and lower noise than CT or ultrasound images. Moreover, MRI images are effective for representing and segmenting micro-soft tissues such as fat and fibrous granular variants in the breast.

**Figure 1:**
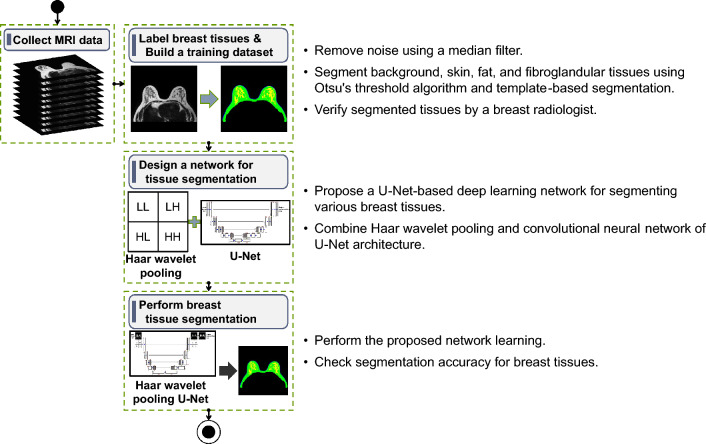
Breast tissue segmentation process based on deep learning network.

Figure 1 shows the breast tissue segmentation process based on a deep learning network for breast shape reconstruction. In the first step, MRI images in Digital Imaging Communication in Medicine (DICOM) format were collected. In the second step, breast tissues from collected MRI breast images were labeled to build a dataset for training the deep learning network. Labeling steps included removing noise from the MRI image with a median filter using Otsu's threshold algorithm, expanding to all MRI images through the template-based segmentation method based on the segmented tissues, and verifying labels by a radiologist. In the third step, U-Net based on Haar wavelet pooling was designed to segment breast tissues for breast shape reconstruction. This study combined U-Net with Haar wavelet pooling to improve the multi-class semantic segmentation performance for MRI breast images. The U-Net based on Haar wavelet pooling uses the LL sub-band, which holds an approximate value of the input image, and three distinct frequency sub-bands (LH, HL, and HH), which have detailed edge features. Therefore, breast tissue features could be effectively extracted by reducing the loss of image information in the subsampling. The U-Net based on Haar wavelet pooling was trained with constructed datasets, and its performance was then tested.

### Building an MRI dataset for breast tissue segmentation

A set of data, including labels for every pixel of the MRI data, is required for breast tissue segmentation with deep learning. In this study, an MRI dataset was collected from the breast diagnosis database^26^ of The Cancer Imaging Archive (TCIA), an open-access database for medical images for cancer research. The breast diagnosis database contains medical images of breast cancer patients as well as cases of breast diagnosis such as high-risk normal, DCIS, fibroids, and lobular carcinomas. Each image was captured with three pulses (T2, STIR, BLISS) using a Phillips 1.5 T MRI system. Breast MRI images of 89 breast cancer patients were obtained at 2 mm intervals at resolutions of 500 to 600 DPI, with 80 to 90 MRI slices per person. This study used MRI slice images of T2-weighted pulse sequences data.

**Figure 2:**
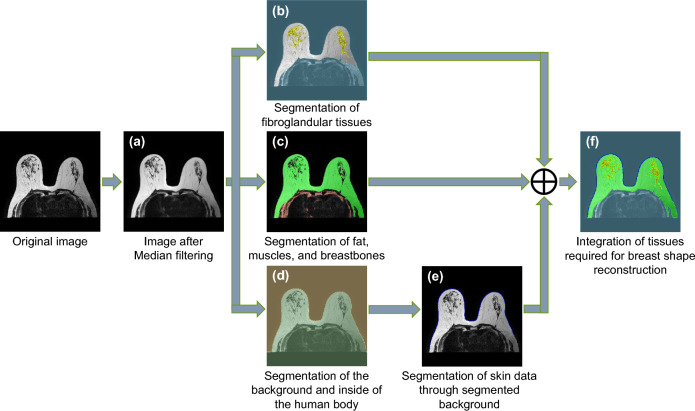
The step-by-step process for breast tissue labeling.

Figure 2 shows a step-by-step process of labeling breast tissues from MRI slice images. The breast tissue should be segmented into skin, fat, fibro-glandular tissue, and background because the upper part of the pectoral muscle is incised in a mastectomy. MRI images often suffer from the presence of salt and pepper noise, which is a type of impulse noise that appears as random white or black dots and can distort the image. To address this issue, we employed a median filter, which is known to be effective in removing point noise. Specifically, we applied the median filter to the slice image, as illustrated in Fig. 2a, to eliminate the salt and pepper noise and improve the image quality. As a result, we were able to obtain more accurate and reliable data for further processing and analysis. Otsu's threshold algorithm was used to segment breast tissues from denoised MRI images. This algorithm could separate the foreground and background by a threshold based on the distribution of pixels in the image. Multiple thresholds were utilized to separate breast tissues with the same pixel distribution value.

Figure 2b shows the result of segmenting fibro-glandular tissues (yellow region) and the background (light blue region) using Otsu’s threshold algorithm by setting the background and the foreground (fibro-glandular tissues). Figure 2c shows the result of segmenting fat (green region) and the rest of the breast tissues (red region) through Otsu’s threshold algorithm by setting the background and the foreground (fat, muscle, and chest wall). Figure 2d shows that the background (brown region) and the inside of the human body (green region) are separated through Otsu’s threshold algorithm. Skin data are lost during T2-weighted pulse sequence images. Hence, boundary lines between green and brown regions were offset by pixels with a thickness of 2 mm that matched the thickness of the human body's skin, and the result was used as skin data. These segmented skin data were validated through BLISS MRI images of breast cancer patients. In this process, breast-diagnosis cases such as fibroma and breast cancer were recognized and segmented as fibro-glandular tissues because they were diseases expanded by the action of hormones on mammary glands. Breast tissues obtained in the previous step were integrated, as shown in Fig. 2f. Light blue, blue, green, and yellow regions represented the background, skin, fat, and fibro-glandular tissues, respectively.

**Figure 3:**
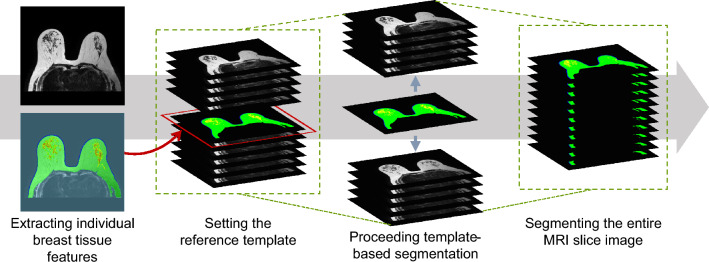
Segmentation of full MRI slice images through template-based segmentation.

We used template-based segmentation to reduce the manual labeling of breast tissues. Template-based segmentation expands with a single MRI slice as a reference template for the remaining other MRI slices; It can extract individual breast tissue features after analyzing each tissue's location, size, shape, and pixel distribution values with cross-sectional MRI slices into which breast tissues are segmented and set as a reference template. The full MRI image in which breast tissues are finally segmented can be obtained by extracting breast tissues with similar characteristics from consecutive MRI slices. Figure 3 shows the process of segmenting the entire MRI slice image through template-based segmentation. T2-weighted pulse sequence data that were input for breast tissue segmentation and the segmented breast tissue are output at 800 × 800 DPI resolution. These labeled data with template-based segmentation were validated by a radiologist.

### Designing U-Net based on Haar wavelet pooling

This study proposed a U-Net based on Haar wavelet pooling in the subsampling stage. The wavelet transform, which has information on the spatial and frequency domains, is expressed as a vibration with an average of zero that vibrates while repeating increase and decrease within a preset time. Wavelet transform can effectively detect sudden signal changes because it describes regional features and provides a signal analysis at different scales and levels. The wavelet transform for a signal $x(t)$ is defined by Eq. (1)^27^:

1 $${W}_{a}x(b)= \frac{1}{\sqrt{a}}\underset{-\infty }{\overset{+\infty }{\int }}x\left(t\right){\Psi }^{*}\left(\frac{t-b}{a}\right)dt\,a>0$$

where $a$ is the parameter for scale change, $b$ is the displacement rate, and ${\Psi }^{*}(t)$ is a continuous basis function called the mother wavelet. The two-dimensional (2D) discrete Haar wavelet transform equation is utilized to compute matrices in deep learning networks, allowing the extraction of high and low frequencies from images. The input image is divided into small image patches, enabling the extraction of high- and low-frequency components through filtering in each image patch. As a result, small image patches are divided by the size of a power of 2, allowing for efficient computation during conversion. This approach enables the preservation of critical image information while reducing computational complexity, minimizing the computational requirements when the Haar wavelet transformation is employed for image segmentation in deep learning networks. In the decomposed image, high-frequency components correspond to various edges and noise, while low-frequency components represent the general information of the input image, such as directionality.

In this study, a 2D discrete Haar wavelet transform that could minimize the computation when converting MRI breast images in the deep learning network was used.

**Figure 4:**
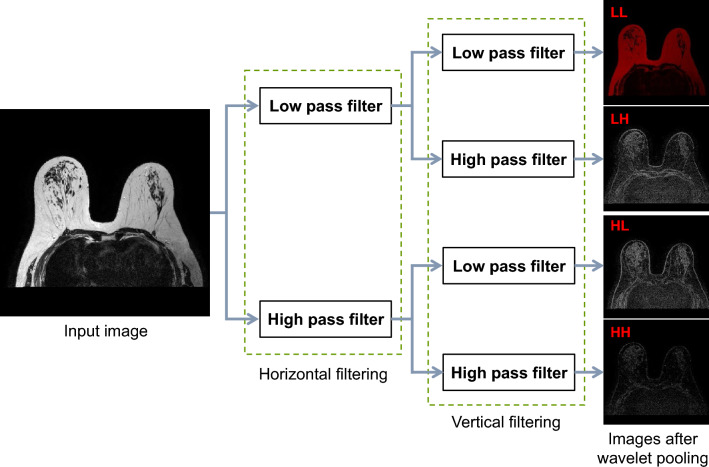
Two-dimensional wavelet pooling structure.

The 2D-wavelet transform presents the input image as a matrix of two-dimensional signals based on the brightness of pixels. Data passing through the 2D-wavelet transform were divided into four bands according to the applied filter. Figure 4 shows the structure of wavelet pooling. The wave pooling process, which utilizes the wavelet transform, involves two distinct steps. Input data were decomposed through a high pass filter and a low pass filter at each step. The size of the input data was reduced because down-sampling was performed in each step. In the first step of wavelet pooling, the input image was horizontally separated into low (L), which was a low-frequency component, and high (H), which was a high-frequency component by applying the horizontal filter. In this process, the approximate value of the input image was decomposed for the low-frequency component, and the detailed value was decomposed for the high-frequency component. In the second step where the vertical filter was applied, images of low- and high-frequency components were vertically separated again and decomposed into four sets of data: LL, LH, HL, and HH bands. Resolutions of data in all bands were reduced to half the resolution of the input data. Data of the LL band had a low-frequency component, indicating the overall trend data of the input data. Data of LH, HL, and HH bands had edge features for vertical, horizontal, and diagonal components, respectively. The segmentation of MRI images requires accurate recognition and differentiation of features based on the orientation of the breast tissue. We utilized four sub-bands generated in the Haar wavelet pooling process to achieve this directionality. Incorporating these sub-bands into deep learning networks can enhance segmentation performance by extracting diverse features from the input data.

U-Net consists of a contracting path that extracts features from the training data and an expansive path for restoring the original resolution. The contracting path performs down-sampling by setting the stride size of the convolution to two in each step, whereas the expansive path performs up-sampling using transposed convolution. The max pooling in the subsampling stage used in previous studies was difficult to generalize because it was sensitive to the overfitting of the dataset^28^. Although some studies have attempted to solve the vanishing gradient problem by passing the information in the contraction path to the expansive path through a skip connection, overfitting still occurs^29^. Breast tissues are delicate data linked by small pixels. Therefore, if max pooling is used, information on the breast tissues might be lost. This study designed a deep learning network of the U-Net architecture based on Haar wavelet pooling for subsampling to segment breast tissues.

Haar wavelet pooling offers the advantage of preserving image directionality, which is invariant to position, scale, and rotation, while reducing spatial information. This enables the recognition features of detailed patterns or boundaries and helps deep learning networks to generalize effectively, making them robust against overfitting^30^. Preservation of orientation information is crucial in medical imaging, where it is necessary to accurately detect and distinguish the characteristics of various tissues and structures based on orientation. Haar wavelet pooling facilitates accurate semantic segmentation and diagnosis in medical images by reducing spatial information while preserving the directional information of these images. Preservation of directional information enables more accurate recognition of subdivided patterns, textures, and borders, thereby improving analysis accuracy.

**Figure 5:**
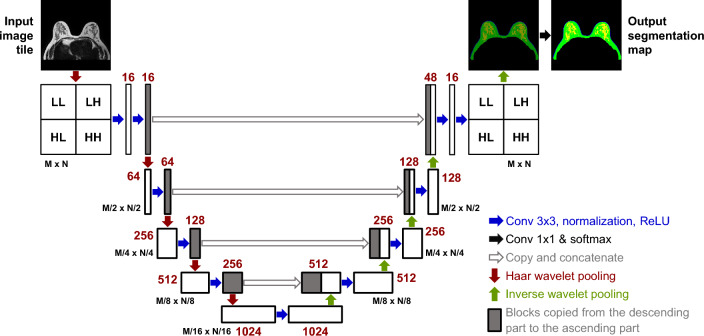
U-Net based on Haar wavelet pooling.

Figure 5 shows the deep learning network architecture that combines Haar wavelet pooling with U-Net. The deep learning network was composed of 12 convolution layers, 5 Haar wavelet pooling layers, and 5 inverse wavelet-based up-sampling layers. Input breast image data were converted into LL, LH, HL, and HH band data by Haar wavelet pooling. These converted data were then transmitted to the convolution layer. The resolution was restored using an inverse wavelet, which could reconstruct data using the output value of wavelet pooling. In the proposed architecture, a batch normalization function and a ReLU activation function were used with each convolution layer. The amount of computation for the network was reduced compared with previous studies by applying the Haar wavelet pooling. The number of existing channels was maintained because the pooling result did not affect the number of channels in the deep learning network. However, the number of input channels of the convolution layer was increased by a factor of four because the U-Net based on Haar wavelet pooling simultaneously used LL, LH, HL, and HH bands.

## System implementation and experimental evaluation

### Implementation environment

Table 3 shows the implementation environment for building the U-Net based on wavelet pooling. The U-Net was executed on Ubuntu Linux. It was implemented in Python using Anaconda, a math and science library, PyTorch, a deep learning library, and CUDA and CuDNN for GPU operation.

**Table 3:** Implementation environment.

Item	Usage	Version
Ubuntu	Operating system	16.04.5 LTS-64bit
Python	Development language	3.8.12
Anaconda	Math and science library	4.10.1
PyTorch	Deep learning library	1.10.0
CUDA	GPU parallel computing library	11.3
CuDNN	GPU-accelerated library	8.2.4

Two NVIDIA Quadro RTX 5000 16G GPUs were interconnected for distributed data-parallel processing for deep learning operations. The interface module was implemented with the DistributedDataParallel library from PyTorch to synchronize IDs of GPU operation processes performed on two graphic cards.

### Experiment and evaluation

Segmentation accuracies for background, skin, fat, and fibro-glandular tissues were analyzed to evaluate the performance of the U-Net based on Haar wavelet pooling. The dataset of 5202 images was divided into a training dataset, a validation dataset, and a test dataset at a ratio of 8:1:1. These data were rotated at a random angle for augmentation of the dataset in the deep learning network training process.

Multi-class semantic segmentation was performed using the U-Net based on Haar wavelet pooling. The resolution of the training data was set to be 800 × 800 DPI, with a batch size of 8, epoch of 200, and learning rate of 0.002. Furthermore, focal loss and adaptive moment estimation optimizer (Adam) were applied. The loss function was compared with cross-entropy, dice loss, and focal loss to find the optimal parameter. Max pooling, average pooling, and Haar wavelet pooling were applied in this experiment to prove the effectiveness of Haar wavelet pooling with subsampling. The segmentation performance was measured using Intersection over Union (IoU) commonly used as a performance evaluation index for segmentation, mIoU (the average of all IoU values), and pixel accuracy. Equations for IoU (2), mIoU (3), and pixel accuracy (4) are shown as follows:

2 $$IOU= \frac{TP}{TP+FP+FN}$$

3 $$mIOU= \frac{1}{k}{\sum }_{i=0}^{k}\frac{TP}{TP+FP+FN}$$

4 $$Pixel \, accuracy= \frac{TP+TN}{TP+TN+FP+FN}$$

where *TP*, *TN*, *FP*, *FN*, and *k* represent true positive, true negative, false positive, false negative, and the number of classes, respectively.

Table 4 shows IoU, mIoU, and pixel accuracy results for the background, skin, fat, and fibro-glandular tissues of the test dataset. Haar wavelet pooling showed higher breast tissue segmentation performance than max pooling and average pooling in the same experimental environment. Furthermore, the deep learning network using focal loss and Haar wavelet pooling showed the highest mIoU and pixel accuracy values. The deep learning network using focal loss and Haar wavelet pooling confirmed that segmentation accuracies for skin and fibro-glandular tissues were relatively high. This is because deep learning networks can be trained effectively because Haar wavelet pooling can reduce the influence of easy negative examples such as background and fat while focusing on training hard negative examples such as skin and fibro-glandular tissues. By contrast, the deep learning network using both dice loss and average pooling showed low segmentation performance. Haar wavelet pooling has been shown to outperform Max pooling and Average pooling in medical image segmentation, particularly in breast tissue images. This is because Haar wavelet pooling can preserve important image features and structure more efficiently than the other two pooling methods, allowing for more accurate segmentation. In breast tissue images, there are both easy negative examples, such as fat and background, and hard negative examples, such as skin and fibro-glandular tissue. Haar wavelet pooling can effectively reduce the influence of easy negative examples while enhancing learning for hard negative examples, resulting in significantly higher segmentation accuracy.

**Table 4:** Experimental results obtained with a combination of training parameters of the deep learning network for breast tissue segmentation.

Pooling method	Loss function	Background IoU (%)	Skin IoU (%)	Fat IoU (%)	Fibro-glandular tissue IoU (%)	mIoU (%)	Pixel accuracy (%)
Max pooling	Cross entropy	98.78	75.44	94.62	76.28	86.28	99.29
Average pooling	Cross entropy	98.39	70.03	93.88	73.15	83.86	99.12
Haar wavelet pooling	Cross entropy	98.80	75.37	94.70	77.66	86.63	99.31
Max pooling	Dice loss	98.62	72.07	94.17	75.52	85.09	99.20
Average pooling	Dice loss	98.40	74.65	93.84	63.94	82.71	98.78
Haar wavelet pooling	Dice loss	98.77	73.63	**94.79**	76.20	85.85	99.29
Max pooling	Focal loss	**98.90**	**76.57**	94.78	**78.58**	**87.21**	**99.34**
Average pooling	Focal loss	98.85	75.47	94.38	73.79	85.62	99.29
Haar wavelet pooling	Focal loss	**98.90**	**77.14**	**94.81**	**79.06**	**87.48**	**99.35**

Significant values are in bold.

Table 5 compares the results of breast tissue segmentation accuracy between the proposed network and previous studies. The IoU, mIoU, and pixel accuracy values for background, skin, fat, and fibro-glandular tissues were measured in this experiment. The U-Net based on Haar wavelet pooling achieved a mIoU of 87.48 and a pixel accuracy of 99.35% for breast tissue segmentation. The Haar wavelet pooling approach is constrained by the accuracy of skin and fibrous tissue, which is lower than that of other breast tissues. To address this issue, our future research endeavors will concentrate on enhancing the accuracy of skin and fibrous tissue. We plan to achieve this through the application of supplementary data augmentation techniques aimed at improving segmentation accuracy or by utilizing loss functions that prioritize specific tissue regions.

**Table 5:** Comparison of complexity, segmentation accuracy, and pixel accuracy values of deep learning networks.

Method	# of params (million)	Background IoU (%)	Skin IoU (%)	Fat IoU (%)	Fibro-glandular tissue IoU (%)	mIoU (%)	Pixel accuracy (%)
U-Net	31.04	98.85	**74.34**	94.20	76.46	85.96	99.28
End-to-end U-Net	27.91	98.85	74.16	93.66	71.99	84.67	99.23
RDA-U-Net	18.85	98.61	75.71	93.89	68.90	84.28	99.19
VEU-Net	14.22	98.63	74.43	94.03	69.77	84.21	99.04
nnU-Net	47.88	**98.86**	73.00	**94.78**	**78.65**	**86.32**	**99.31**
LL wavelet U-Net	21.98	97.00	68.30	90.75	43.67	74.95	98.54
Ours	**24.28**	**98.90**	**77.14**	**94.81**	**79.06**	**87.48**	**99.35**

Significant values are in bold.

The Haar wavelet transformation offers the capability to minimize computational complexity by decomposing the input image into multiple layers. Therefore, by utilizing Haar wavelet pooling in deep learning networks for segmenting MRI breast images, high segmentation accuracy can be achieved with minimal computation, even for high-resolution medical images. Additionally, compared to existing deep learning networks, Haar wavelet pooling offers the advantage of reduced time in medical image analysis and high accuracy.

As demonstrated in Table 5, the proposed method exhibits a performance improvement of 1.16% in mean Intersection over Union (mIOU) relative to previous study^31^. Although the enhancement in mIOU across all tissues is not substantial, our study primarily targeted micro-tissues, such as skin and fibroglandular tissue. Specifically for skin, we observed an improvement of 4.14% when compared to previous study^31^. These findings imply that Haar wavelet pooling plays a significant role in enhancing the recognition of micro-tissues.

### Verification of segmentation results through visualization

**Figure 6:**
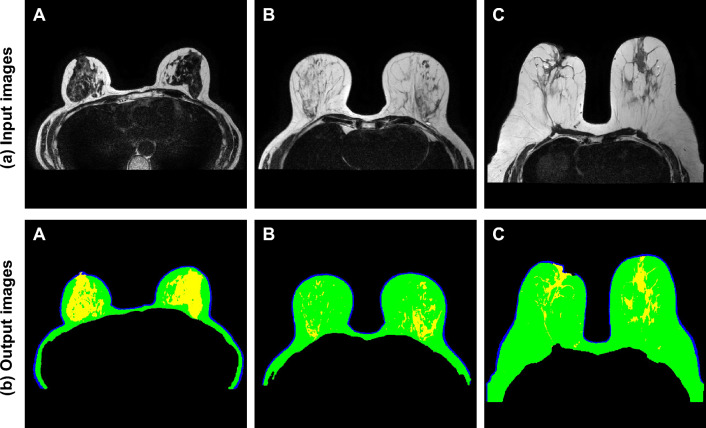
Original MRI images (top) and breast tissue images segmented through the U-Net based on Haar wavelet pooling (bottom) for three female patients.

Figure 6 shows original MRI images of three patients (A, B, and C) with different breast shapes and fibro-glandular tissue densities in the test dataset and images of breast tissues segmented by the proposed network. The top of Fig. 6 depicts the original MRI images and the bottom shows breast tissue images segmented using the proposed network. Black, blue, green, and yellow indicate the background, skin, fat, and fibro-glandular tissues, respectively. For Patient A, a small breast shape and a high density of fibro-glandular tissues were observed. Patient B was characterized by a medium-sized breast shape and low-density fibro-glandular tissues close to the chest wall muscle. Patient C, with a large breast shape, was characterized by moderately dense fibro-glandular tissues. These results showed that the proposed network could effectively segment skin, fat, and fibro-glandular tissues even when MRI images with different breast shapes and fibro-glandular tissue densities were used as input.

**Figure 7:**
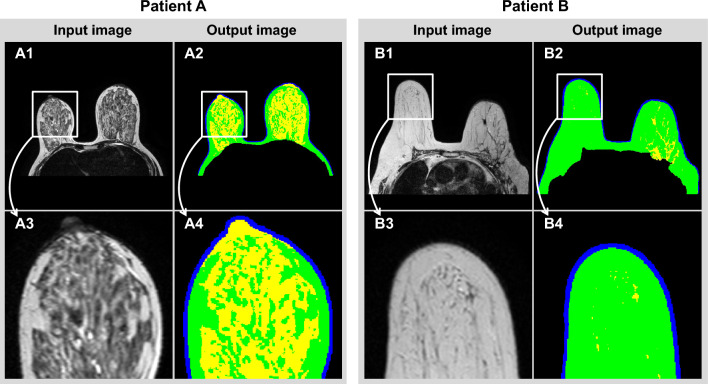
MRI images and breast tissue segmentation images of Patient A with a high segmentation accuracy (mIoU: 90.28) and Patient B with a low segmentation accuracy (mIoU: 77.94).

Figure 7 shows MRI images and segmentation results for two patients. Figure 7 (Patient A) shows the 69th patient out of 89 patients, with a mIoU of 90.28. Figure 7 (Patient B) shows an MRI image of the 58th patient with a mIoU of 77.94. As shown in (A3) and (B3) rectangles in Fig. 7, the fibro-glandular tissue of Patient A had a higher density than that of Patient B. As for Patient B, with a low density of fibro-glandular tissues, the mIoU of the segmented breast tissue was lower than that of Patient A with a high density of fibro-glandular tissues. This observation indicated that the U-Net based on Haar wavelet pooling could effectively segment breast images of women with a high density of fibro-glandular tissues.

**Figure 8:**
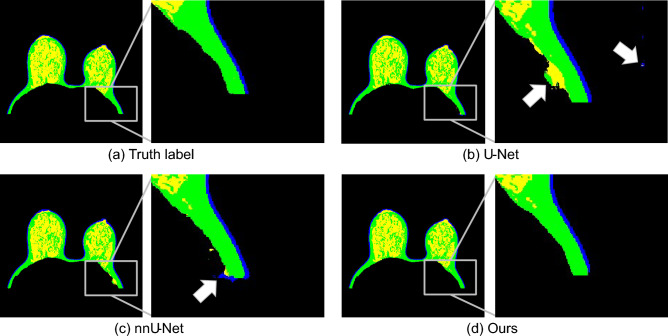
Comparison of breast tissue images segmented by three deep learning networks with the highest mIoU values.

Figure 8 visualizes the ground truth image and the segmented breast tissue image with U-Net, nnU-Net, and U-Net based on Haar wavelet pooling. The rectangle area indicated that the U-Net based on Haar wavelet pooling segmented breast tissues more accurately than U-Net and nnU-Net. By contrast, in Fig. 8b, the outside of the background was misrecognized as skin tissue and the inside as fibro-glandular tissue and fat, owing to the noise of the background. When Fig. 8b, showing an image of breast tissue segmented through nnU-Net, was compared with the ground truth, the background was incorrectly segmented into fibro-glandular tissue, skin, and fat because of the noise inside the background. These results showed that the proposed network could distinguish the noise of the input image and the breast tissue more accurately than methods described in previous studies and accurately segment delicate soft tissues and skin of the mammary gland. As depicted in Fig. 8b and c show misclassifications of fat and fibroglandular tissues at the lower regions. However, these tissues are accurately identified in the proposed method. By utilizing the directional information from the four sub-images, Haar wavelet pooling aids image segmentation, thereby preventing potential noise that could arise in adjacent tissues. This, in turn, yields improved results.

### Consent to participate

All authors consent for participation.

## Conclusion

Recently, deep learning networks with excellent performance have been introduced in various studies for medical image segmentation. Many methods have been proposed for segmenting breast tissues using binary-image segmentation to diagnose breast diseases such as breast cancer and breast tumors. However, conventional methods are time-consuming and labor-intensive because the ROI for breast tissues is manually set and the quality of the segmented tissue varies depending on the skill level of the worker. To address this issue, we proposed a U-Net based on Haar wavelet pooling for multi-class semantic segmentation of breast tissues from MRI images. In addition, a labeled dataset was built to train the network for breast shape reconstruction. The proposed network achieved a mIoU of 87.48 and a pixel accuracy of 99.35%. In particular, the network accurately segmented breast tissues of women with a high density of fine mammary glands.

It is quite challenging to identify an effective method for substantially enhancing the IOU value in medical image data segmentation. Nevertheless, our method was able to make a modest improvement to the IOU value, which will have a significant impact on breast shape reconstruction. While the method proposed in this paper concentrates on 2D image segmentation, we plan to explore the possibility of extending it to 3D image segmentation in future studies. We aim to compare its performance against established 3D segmentation methods like 3D U-Net. The findings of this comparison will help us broaden the application scope of the proposed method. Further, we aim to enhance its segmentation accuracy by considering the inter-slice correlation in MRI images.

## Data Availability

The datasets used and/or analysed during the current study available from the corresponding author on reasonable request.
